# Psychometric testing of the mental health inventory in an Arabian context: Cross‐cultural validation study

**DOI:** 10.1002/nop2.149

**Published:** 2018-05-09

**Authors:** Abbas Al Mutair, Mohammed Al Mohaini, Ritin Fernandez, Lorna Moxham, Samuel Lapkin, Wilma ten Ham‐Baloyi

**Affiliations:** ^1^ Inaya Medical College Riyadh Saudi Arabia; ^2^ King Abdullah International Medical Research Center King Saud bin Abdulaziz University for Health Sciences CAMS‐HS MNGHA Alahsa Saudi Arabia; ^3^ School of Nursing Faculty of Science Medicine & Health University of Wollongong Wollongong NSW Australia; ^4^ Centre for Research in Nursing and Health St George Hospital Kogarah NSW Australia; ^5^ Faculty of Science Medicine & Health School of Nursing University of Wollongong Wollongong NSW Australia; ^6^ Faculty of Health Sciences Nelson Mandela Metropolitan University Port Elizabeth South Africa

**Keywords:** emotional well‐being, Mental Health Inventory, nursing, psychometric properties, Saudi Arabia

## Abstract

**Aim:**

The aim of this study is to establish a valid and reliable culturally adapted instrument which can be used in the Arabian context to measure emotional well‐being.

**Design:**

The Mental Health Inventory tool was used to investigate the emotional well‐being of Saudi nursing students. The instrument was originally developed in English and in a western cultural setting. As such, there was a need to translate and validate the instrument in Arabic for use in the Arabian setting.

**Methods:**

The Arabic version MHI 38 (AV‐MHI‐38) was translated, reviewed and revised, then evaluated with a sample of Arabic‐speaking nursing students from Saudi Arabia using cross‐sectional multicentre survey approach. An iterative forward‐backward‐forward sequence of item translation and review by a bilingual and bicultural expert panel was then completed. The psychometric properties of the AV‐MHI‐38 were examined through an exploratory factor analysis, confirmatory factor analysis, correlation among factors and reliability assessment.

**Results:**

The sample consisted of 252 nursing students from two different universities located in different geographic regions in Saudi Arabia. The mean age was 20.93 years, made up of 230 female and 22 male participants. An a priori two‐factor model showed satisfactory fit with modifications. Results indicated two component AV‐MHI‐38 with 46.09% of the total variance and excellent internal consistency. The AV‐MHI‐38 had good psychometric properties and the two subscales had good internal consistency with Cronbach's and acceptable reliability measures. The AV‐MHI can be used to assess emotional well‐being among Arabic‐speaking populations of nursing students, nurses and other healthcare providers. The instrument can be used to identify the emotional well‐being of students and initiating strategies to support them to decrease their study and work‐related stress, anxiety and depression.

## 1 INTRODUCTION

Nursing students are considered more at risk of developing psychological distress than students in other university programs of study. Psychological distress is a negative state of emotions and can manifest in symptoms of depression and anxiety (Drapeau et al., 2010). There is a growing body of research evidence that suggests nursing students may experience mental health issues (Lo, 2002; Wolf, Stidham, & Ross, 2015). Understanding and identifying nursing students' emotional well‐being can therefore instigate strategies to mitigate negative outcomes for this cohort.

### 1.1 Background

The availability of valid measures of emotional and psychological well‐being that are culturally inclusive for cohorts other than those who speak and read English is important for research in mental health outcomes among them. Such measures can be used to establish levels of emotional well‐being and can contribute to ascertaining the mental health of those completing the instrument (Terjesen, Salhany, & Sciutto, 2009). One such measure is the Mental Health Inventory (MHI‐38) which has been widely used in many studies to examine the emotional well‐being of different populations, but thus far, only in English and Chinese. There is a need therefore to contribute to the body of knowledge regarding the emotional well‐being of nursing students in countries other than those who speak and read English.

Nursing education in Saudi Arabia is conducted in English, but English is a complex language full of contradictions and conundrums compared with Arabic and as such there are several factors and issues affecting English Language education in Saudi Arabia (Al Mutair & Redwan, 2016). Almost half of the nursing students surveyed in a study identified English proficiency as one of the most challenging components of studying nursing (Al Mutair & Redwan, 2016). Given the difficulties with language‐based grammatical issues, a cross‐cultural adaptation and cross‐validation of the MHI instrument were considered important for mental health research. Beaton, Bombardier, Guillemin, and Ferraz (2000) suggested that the term—“cross‐cultural adaptation” is used to encompass a process that looks at both language translation and cultural adaptation issues in the process of preparing a questionnaire to be used in another setting. Attention to the questionnaire details allows increased confidence and maximizes the attainment of semantic, idiomatic, experiential and conceptual equivalence between the source and target questionnaires (Sousa & Rojjanasrirat, 2011). This is important to capture accurate data and to ensure cultural inclusivity. The aim of this study therefore was to ensure that the valid and reliable tool known as the MHI‐38 was appropriately culturally adapted and able to be used in an Arabian context to measure the emotional well‐being of nursing students.

## 2 METHOD

### 2.1 Design

A cross‐sectional multicentre survey study was used to collect data through the use of the Arabic version of the Mental Health Inventory (AV‐MHI‐38). The development of the original MHI‐38 was grounded as part of the National Health Insurance Study (Veit & Ware, 1983) and has been used extensively in a variety of populations and there is considerable evidence for its validity (Heubeck & Neill, 2000; Manne & Schnoll, 2001; Veit & Ware, 1983). The MHI‐38 was developed and psychometrically tested in English‐speaking countries and consists of 38 items with two global scales of Psychological Distress (24 items) and Psychological Well‐being (14 items).

The Mental Health Inventory (MHI‐38) was translated from English into Arabic using well‐established methodological approaches for translating, adapting and validating instruments (Beaton et al., 2000; Sousa & Rojjanasrirat, 2011). The first step involved independent forward or one‐way translation of the MHI‐38 English version into Arabic by one bilingual translator who was fluent in both English and Arabic. After it was translated into Arabic, the Arabic version was then given to a competent bilingual person to back‐translate into English. As a result, there were two versions in English, the original and the back‐translated version and one in the target language, Arabic. The next phase was the English and Arabic versions were both given to an independent bilingual professional English teacher who was specially recruited for the task to read, check and confirm the Arabic translation of the documents and to check the equivalent versions of English and Arabic to see whether they measured the same constructs.

An expert panel comprising of eight researchers and academicians who spoke both English and Arabic fluently and had tertiary qualifications was established. The panel members were asked to rate the instructions and items of the Arabic versions of the MHI‐38 using a dichotomous scale (1 = clear or 2 = unclear). They were also asked to provide suggestions as to how to word the statements or items identified as unclear, so as to ensure they had greater clarity. The item level and overall content validity indexes (CVI) were calculated for the AV‐MHI‐38 and a CVI of 0.78 or higher was acceptable (Polit & Beck, 2004).

### 2.2 Sample and setting

A probability convenience sample was used to recruit second, third and fourth year nursing students for the study from a government university and a private college in Saudi Arabia. A sample size of 200 participants was determined to allow estimation of effects of moderate size at 5% significance level with 80% power. The actual sample size in the study reported in this paper was 252 participants.

### 2.3 Data collection

Data were collected between December 2016 ‐ February 2017. Participants completed the questionnaire which gathered data related to age, gender, marital status, place of living, year of study and employment status and the AV‐MHI‐38 instrument. No names or any other identifiable information were collected.

### 2.4 Ethical consideration

Participants were fully informed that their participation is voluntary. Ethical approval to conduct the study in Saudi Arabia was obtained from the Ethics Committee of the participating university (No: IRBC/1121/16) and college (No: IRB/01/0117). Written instruction about the study was given to the study participants by a research assistant. Students were informed that completion of the survey implied their consent for participation.

### 2.5 Data analysis

All data were entered in Excel and transferred into IBM SPSS Statistics, version 22 and AMOS version 22 (IBM Corp, 2013) for analysis. The demographic data were summarized using descriptive statistics. The psychometric properties of the AV‐MHI‐38 were investigated using a cross‐validation approach involving both Confirmatory factor analysis (CFA) and exploratory factor analysis (EFA) (DeVellis, 2016). First, the sample (*N* = 252) was randomly split using a divergence procedure in SPSS (IBM Corp, 2013) into two subsamples of approximately 50% of the cases. Confirmatory factor analysis (CFA), using the first subsample (*N* = 123) was undertaken to verify the AV‐MHI‐38 factor structure with the original 38‐item, two‐factor structure model (Veit & Ware, 1983). The recommendation provided by Hu and Bentler (1999) was used to evaluate adequacy of the CFA model using the following cut‐off values: the ratio of chi‐square to its degree of freedom; Goodness of Fit index (GFI) ≥0.90; the Root Mean Square Error of Approximation (RMSEA) <0.008; the Comparative Fit Index (CFI) >0.90; and the Standardized Root Mean Square Residual (SRMR) <0.05.

The results of the CFA suggested room for improvement in model fit; therefore, an EFA was conducted using the second subsample (*N* = 129) that was not used for CFA. Principal component factor (PCA) extraction with orthogonal varimax rotation was used as applied in the original study (Veit & Ware, 1983) and other similar studies (Marques, Pais‐Ribeiro, & Lopez, 2011) to determine if the original two‐factor structure could be replicated in this sample. The number of potential factors to retain was determined using four criteria, namely; Kaiser's criteria (eigenvalue >1 rule) (DeVellis, 2016); scree plot (Williams, Onsman, & Brown, 2012a); cumulative percent of variance extracted; and parallel analysis (Williams, Onsman, & Brown, 2012bb). The decision to consider loadings of 0.30 or higher as significant was made a priori (Kline, 2013). The reliability of the scale and subscales were assessed using Cronbach's alpha coefficient (Streiner, Norman, & Cairney, 2014).

## 3 RESULTS

### 3.1 Content validity

The CVI scores for the individual AV‐MHI‐38 items ranged from 0.63 to 1.0 and was 0.85 for the entire questionnaire. Minor changes were made based on suggestion from the expert panel. A final version of the translated MHI in Arabic was derived and named “Arabic Version of Mental Health Inventory‐38 (AV‐MHI‐38)”.

### 3.2 Demographics

Of the 350 surveys that were disseminated, completed surveys were received from 252 respondents for a response rate of 72%. The majority (91.3%) of the participants were female and the ages of the participants ranged from 18 to 38 years, with an average age of 20.93 years (*SD* 3.15). Only 28 (11.1%) of the participants were employed and the majority (94%) lived with their family. The composition of the study sample was very similar to that of the population of nursing students in Saudi Arabia in terms of age, gender, marital status, living arrangement and employment status (Al Mutair & Redwan, 2016). The demographic characteristics of the sample are shown in Table 1.

**Table 1:** Demographic characteristics of the participants (*N *=* *252)

Demographic characteristic	Frequency	%
Campus location		
Site 1	47	18.7
Site 2	205	81.3
Year of study		
Year 2	184	74.5
Year 3	43	17.4
Year 4	16	6.5
Gender		
Female	230	91.3
Male	22	8.7
Marital status		
Single/Divorced	205	82.0
Married/living with a partner	45	18.0
Living arrangement		
With the family	235	94.0
On campus	2	0.8
Off campus	13	5.2
Employment status		
Employed	28	11.1
Not employed	224	88.9

### 3.3 Validation of the Arabic version of Mental Health Inventory 38

The a priori two‐factor model fit the data poorly. Model fit indices as suggested by Hu and Bentler (1999); χ2, (*df* = 661) = 1064.44, *p* < .001, GFI = 0.797, RMSEA = 0.071 (PCLOSE < 0.001), CFI = 0.797 and SRMR = 0.805, indicates that the factor structure found in the original MHI‐38 (Veit & Ware, 1983) was not replicated in this study sample. An inspection of the CFA output and modification indices identified six items that had low factor loadings (<0.30). These were: item 3‐ “How often did you become nervous or jumpy when faced with excitement or unexpected situations during the past month?”; item 15‐ “During the past month, how often did your hands shake when you tried to do something?”; item 16‐ “During the past month, how often did you feel that you had nothing to look forward to?”; item 21‐ “During the past month, how often have you felt that others would be better off if you were dead?”; item 28‐ “During the past month, did you think about taking your own life?”; and item 35‐ “How often during the past month did you find yourself trying to calm down?”. However, allowing covariance between identified items, inclusion of the error of covariance and removing items with low factor loadings failed to improve the model fit. These results of the CFA were not informative with respect to the structure of the AV‐MHI, subsequently, exploratory factor analysis was used as a data‐driven approach to generate questionnaire subscales.

### 3.4 Construct validity of the AV‐MHI‐38

The Kaiser‐Meyer‐Olkin (1974) Measure of Sampling Adequacy for the 38 items on the second subsample (*N* = 129) was 0.90 and the Bartlett's Test of Sphericity (χ2 = 3043.08 *df* = 703, *p *=* *.001). These results met the recommended values, thereby demonstrating the adequacy of the sample for factor analysis (Pett, Lackey, & Sullivan, 2003). Results of the PCA indicated two components with eigenvalues greater than 1.0 in the unrotated factor matrix, explaining 46.09% of the total variance. An inspection of the scree plot indicated a departure from linearity after the second component and these two were retained for factor rotation. An orthogonal varimax indicated that the two factors had high eigenvalues and all 38 items had loadings of 0.30 or above on the relevant factor. Eighteen items loaded on factor 1 (Psychological Distress) and 20 items loaded on factor 2 (Psychological Well‐being). The two factors solution explained 46.09% of the total variance (Table 2).

**Table 2:** Rotated loading matrix of the EFA using subsample 1 (*N *=* *129)

Item number	F1—loading	F2—loading	Item	Communality	Cronbach's alpha if item deleted
Subscale 1 α = 0.68					
34	**0.77**	−0.28	During the past month, how much of the time were you a happy person?	0.67	0.63
31	**0.75**	−0.30	How much of the time, during the past month, have you felt cheerful, light‐hearted?	0.65	0.63
26	**0.73**	−0.03	During the past month, how much of the time has living been a wonderful adventure for you?	0.53	0.62
7	**0.71**	−0.21	During the past month, how much of the time have you generally enjoyed the things you do?	0.55	0.62
12	**0.69**	−0.20	When you have got up in the morning, this past month, about how often did you expect to have an interesting day?	0.51	0.62
32	−**0.66**	0.55	During the past month, how often did you get rattled, upset or flustered?	0.73	0.76
5	**0.65**	−0.25	How much of the time, during the past month, has your daily life been full of things that were interesting to you?	0.48	0.64
37	**0.64**	−0.25	How often, during the past month, have you been waking up feeling fresh and rested?	0.47	0.63
10	**0.64**	0.17	During the past month, how much of the time have you felt loved and wanted?	0.44	0.65
36	−**0.64**	0.50	During the past month, how much of the time have you been in low or very low spirits?	0.65	0.76
18	**0.64**	‐0.28	How much of the time, during the past month, have you felt emotionally stable?	0.49	0.63
27	−**0.60**	0.53	How often, during the past month, have you felt so down in the dumps that nothing could cheer you up?	0.64	0.76
23	**0.59**	−0.14	How much of the time, during the past month, did you feel that your love relationships, loving and being loved, were full and complete?	0.37	0.64
17	**0.54**	−0.44	How much of the time, during the past month, have you felt calm and peaceful?	0.49	0.63
4	**0.54**	−0.11	During the past month, how much of the time have you felt that the future looks hopeful and promising?	0.31	0.65
22	**0.54**	−0.19	How much of the time, during the past month, were you able to relax without difficulty?	0.32	0.64
1	**0.53**	−0.30	How happy, satisfied or pleased have you been with your personal life during the past month?	0.38	0.65
2	−**0.43**	0.21	How much of the time have you felt lonely during the past month?	0.23	0.73
Subscale 2 α = 0.80					
13	−0.11	**0.77**	During the past month, how much of the time have you felt tense or “high‐strung”?	0.60	0.78
25	−0.28	**0.77**	How much have you been bothered by nervousness, or your “nerves”, during the past month?	0.66	0.77
11	−0.15	**0.74**	How much of the time, during the past month, have you been a very nervous person?	0.56	0.78
33	−0.28	**0.72**	During the past month, have you been anxious or worried?	0.59	0.78
38	−0.39	**0.70**	During the past month, have you been under or felt you were under any strain, stress or pressure?	0.64	0.78
29	−0.24	**0.69**	During the past month, how much of the time have you felt restless, fidgety or impatient?	0.54	0.78
19	−0.45	**0.67**	How much of the time, during the past month, have you felt downhearted and blue?	0.66	0.77
30	−0.38	**0.65**	During the past month, how much of the time have you been moody or brooded about things?	0.57	0.77
3	0.05	**0.64**	How often did you become nervous or jumpy when faced with excitement or unexpected situations during the past month?	0.41	0.79
8	0.35	−**0.63**	During the past month, have you had any reason to wonder if you were losing your mind, or losing control over the way you act, talk, think, feel or of your memory?	0.51	0.85
20	−0.28	**0.61**	How often have you felt like crying, during the past month?	0.45	0.78
9	−0.43	**0.52**	Did you feel depressed during the past month?	0.46	0.79
14	0.31	−**0.52**	During the past month, have you been in firm control of your behaviour, thoughts, emotions or feelings?	0.36	0.84
24	−0.36	**0.50**	How often, during the past month, did you feel that nothing turned out for you the way you wanted it to?	0.38	0.78
15	0.18	**0.50**	During the past month, how often did your hands shake when you tried to do something?	0.28	0.80
6	0.39	−**0.48**	How much of the time, during the past month, did you feel relaxed and free from tension?	0.38	0.84
28	−0.15	**0.38**	During the past month, did you think about taking your own life?	0.17	0.80
16	−0.22	**0.34**	During the past month, how often did you feel that you had nothing to look forward to?	0.16	0.79
35	−0.08	**0.31**	How often during the past month, did you find yourself trying to calm down?	0.10	0.80
21	−0.09	**0.30**	During the past month, how often have you felt that others would be better off if you were dead?	0.10	0.80

Numbers in bold indicate loading of items to their corresponding factor

### 3.5 Exploratory factor analysis results

Internal consistency using Cronbach's alpha (α) coefficient for the AV‐MHI‐38 was 0.58 and improved to 0.62 by removing item 8‐ “During the past month, have you had any reason to wonder if you were losing your mind, or losing control over the way you act, talk, think, feel or of your memory? The value was 0.68 for Factor 1 and could be further improved to 0.76 by removing item 36‐ “During the past month, how much of the time have you been in low or very low spirits?” Factor 2 had a Cronbach's alpha coefficient of 0.80 and this could be improved to 0.85 with the removal of item 8‐ “During the past month, have you had any reason to wonder if you were losing your mind, or losing control over the way you act, talk, think, feel or of your memory?”

## 4 DISCUSSION

The demand for questionnaires for assessing mental health and emotional well‐being across countries and languages is increasing; hence, it is vital that the psychometric properties of instruments are tested prior to use across culturally and linguistically diverse populations (Lai, 2013). The aim of the study was to translate and test the psychometric properties of the MHI‐38 in an Arabian context using data collected from a sample of nursing students in Saudi Arabia. A cross‐validation approach involving both CFA and EFA was used to validate the AV‐MHI‐38.

Results of the CFA did not support the proposed construct of the original two‐factor model even after allowing covariance and inclusion of error covariance. We therefore explored the factor structure of AV‐MHI‐38 using EFA. The results of the EFA revealed that the AV‐MHI‐38 clustered into two separate factors in a manner that is congruent with the original version of the scale (Veit & Ware, 1983). However, item analysis indicated that the AV‐MHI‐38 did not replicate the exact factor structure of the MHI‐38. More specifically, six items (2, 6,18,27,32 and 36) did not load on the factors as per the original MHI‐38. Another key finding is that items 8 (During the past month, have you had any reason to wonder if you were losing your mind, or losing control over the way you act, talk, think, feel or of your memory?) and 36 (During the past month, how much of the time have you been in low or very low spirits?) were identified as good candidates for removal. With these items removed, the alpha reliability coefficient values improved from 0.65 ‐ 0.76 for factor 1 and from 0.80 ‐ 0.85 for factor 2 and a value of 0.85 for the overall AV‐MHI‐38.

Various reasons for this could be postulated these findings. These findings are comparable with those reported in other studies involving the translation, adaptation or psychometric testing of the MHI‐38 (Khan, Hanif, & Tariq, 2015; Liang, Wu, Krause, Chiang, & Wu 1992). For example, a validation study of MHI‐38 involving Chinese in Taiwan reported that a 26 item two‐factor model was the best fit (Liang et al. 1992). A more recent study involving the translation of the MHI‐38 into Urdu language was conducted in Pakistan (Khan et al., 2015). In this Urdu version, a MHI‐38 with 16 items for factor 1 and 22 for factor 2 was reported. Taken in the context of our study, there are likely to be fundamental socio‐cultural differences between the Saudi Arabian participants used to test properties of the AV‐MHI and participants in other countries. These two items identified for removal appear to predominantly assess the frequency or intensity of negative aspects of mental health such as anxiety and depression. This may be in part due to the fact that cultural differences in the experience and communication of emotional well‐being might also have mediated some differences in measurement properties of AV‐MHI‐38 in Saudi Arabia. The results also suggest that older students had greater psychological well‐being and relatively less psychological distress, this again could be culture‐related but warrants further investigation. Culturally, in Saudi Arabia, families have strong emotional ties, having an impact on the psychological well‐being of the respondents. As most students indicated that they lived with their families, this may have had an impact on a greater psychological well‐being of the respondents. This might also suggest that these items may not accurately describe relevant aspects of emotional well‐being among the sample of nursing students in Saudi Arabia. To our knowledge, this apparent sensitivity nature of some of the MHI‐38 items has not been reported previously. This is not surprising considering that qualitative data that is more suited to exploring such anomalies are not routinely collected when validating questionnaires. Further psychometric testing among other samples is therefore needed.

### 4.1 Limitations

There are several limitations which must be considered when interpreting the results of this study. First, we selected a convenience sample from two universities in Saudi Arabia. As such, our results may not generalize to all Saudi Arabian nursing students. Despite this limitation, our large sample size allowed us to randomly select separate confirmatory and exploratory subsamples. The second limitation is that other recommended psychometric properties were not evaluated such as convergent, discriminant and concurrent validity. The overall sample was also over‐represented by second year nursing students as this is the case in the nursing schools in Saudi Arabia; thus, the vast majority of the students studying year one and two. Lastly, given the Arabian culture the responses could have been prone to social desirability bias. Despite these limitations, our findings make meaningful contributes to the body of knowledge in the context of nursing research.

## 5 CONCLUSION

This is the first study to translate and validate the two‐factor model of the MHI‐38 with a sample Saudi Arabian context. There is a paucity of literature concerning psychometric cross‐cultural validation and evaluation of the reliability of the MHI‐38 which restricted the current study's capacity to make comparisons. Therefore, the results of this study and experience gained through this cross‐cultural validation undertaking this process make an important contribution to the Saudi and Arabic contexts.

The AV‐MHI‐38 has concept, content and constructs equivalent to the MHI‐38 English version. The AV‐MHI‐38 can be considered a reliable and valid scale that measures emotional well‐being among Arabic‐speaking participants. The study findings further support the reliability, homogeneity and construct‐related validity of the AV‐MHI‐38 when used to examine emotional well‐being in Saudi Arabian nursing students. The AV‐MHI‐38 psychometric properties are similar to those of other versions of the scale but further psychometric testing is recommended among different populations. The instrument is used to measure emotional well‐being and psychological distress, helping to understand and identify what influences emotional well‐being among Saudi and Arabic nursing. This has the potential to initiate strategies to support students which in turn could help to decrease stress, anxiety and depression.

### 5.1 Implications for nursing and health policy

The findings presented in this study illustrate the potential for using the MHI‐38 in assessing the mental well‐being in culturally and linguistically diverse populations. The instrument can be used to measure the emotional well‐being and psychological distress to help understanding and identify what influences emotional well‐being. This has the potential to initiate strategies to support students which in turn could help to decrease stress, anxiety and depression. Drawing comparisons across various study samples would allow for expanded understanding of the effects of psychological distress among a variety of nursing students. This will in turn inform the development of appropriate interventions aimed at assessing the emotional well‐being among nursing students.

## CONFLICT OF INTEREST

The authors declare there is no conflict of interest.

## AUTHOR CONTRIBUTIONS

AA, FR, ML, TW, LS: Study design. AA, AM: Data collection. AM: Data entry. RF, LS: Data analysis. RF, LM: Study supervision. AA, FR, ML, TW, LS: Manuscript writing.
